# A new *CYP21A1P/CYP21A2 *chimeric gene identified in an Italian woman suffering from classical congenital adrenal hyperplasia form

**DOI:** 10.1186/1471-2350-10-72

**Published:** 2009-07-22

**Authors:** Paola Concolino, Enrica Mello, Angelo Minucci, Emiliano Giardina, Cecilia Zuppi, Vincenzo Toscano, Ettore Capoluongo

**Affiliations:** 1 Laboratory of Molecular Biology, Institute of Biochemistry and Clinical Biochemistry, Catholic University, Largo A. Gemelli 8, 00168 Rome, Italy; 2 Department of Endocrinology, II Faculty of Medicine, University 'La Sapienza', S. Andrea Hospital, Rome, Italy; 3 Centre of Excellence for Genomic Risk Assessment in Multifactorial and Complex Diseases, School of Medicine, Tor Vergata University of Rome, Italy

## Abstract

**Background:**

More than 90% of Congenital Adrenal Hyperplasia (CAH) cases are associated with mutations in the 21-hydroxylase gene *(CYP21A2) *in the HLA class III area on the short arm of chromosome 6p21.3. In this region, a 30 kb deletion produces a non functional chimeric gene with its 5' and 3' ends corresponding to *CYP21A1P *pseudogene and *CYP21A2*, respectively. To date, five different *CYP21A1P/CYP21A2 *chimeric genes have been found and characterized in recent studies. In this paper, we describe a new *CYP21A1P/CYP21A2 *chimera (*CH-6*) found in an Italian CAH patient.

**Methods:**

Southern blot analysis and *CYP21A2 *sequencing were performed on the patient. In addition, in order to isolate the new *CH-6 *chimeric gene, two different strategies were used.

**Results:**

The *CYP21A2 *sequencing analysis showed that the patient was homozygote for the g.655C/A>G mutation and heterozygote for the p.P30L missense mutation. In addition, the promoter sequence revealed the presence, in heterozygosis, of 13 SNPs generally produced by microconversion events between gene and pseudogene. Southern blot analysis showed that the woman was heterozygote for the classic 30-kb deletion producing a new *CYP21A1P/CYP21A2 *chimeric gene (*CH-6*). The hybrid junction site was located between the end of intron 2 pseudogene, after the g.656C/A>G mutation, and the beginning of exon 3, before the 8 bp deletion. Consequently, *CH-6 *carries three mutations: the weak pseudogene promoter region, the p.P30L and the g.655C/A>G splice mutation.

**Conclusion:**

We describe a new *CYP21A1P/CYP21A2 *chimera (*CH-6*), associated with the HLA-B15, DR13 haplotype, in a young Italian CAH patient.

## Background

Congenital Adrenal Hyperplasia (CAH) is an autosomal recessive disorder mainly caused by defects in the steroid 21-hydroxylase gene (*CYP21A2*). Steroid 21-hydroxylase is a microsomal cytochrome P450 required for the synthesis of cortisol and aldosterone but not for the synthesis of sex steroids. The reduced synthesis of cortisol and aldosterone leads to excessive androgen production [1].

CAH includes a wide spectrum of clinical manifestations. Milder forms are referred to as Nonclassic (NC) or late-onset CAH [2]. Patients with the severe classic disease form are classified as either salt-wasting (SW) or simple-virilising (SV) depending on whether or not synthesis of the salt retaining hormone, aldosterone, is affected [3].

The gene encoding 21-hydroxylase, *CYP21A2*, is located in the HLA class III region on the short arm of chromosome 6p21.3 [4]. In this region, four tandemly arranged genes – serine/threonine Kinase *RP*, complement *C4*, steroid 21-hydroxylase *CYP21*, and tenascin *TNX *– are organized as a genetic unit designated as a RCCX module. In a RCCX bimodular haplotype, duplication of the RCCX module occurs and the orientation of genes, from telomere to centromere, is: *RP1-C4A-CYP21A1P-TNXA-RP2-C4B-CYP21A2-TNXB*. The three pseudogenes, *CYP21A1P-TNXA *and *RP2*, located between the two C4 loci, do not encode functional proteins [5,6].

In the Caucasian population, bimodular and monomodular RCCX organizations are present in about 69% and 17% of chromosome 6, respectively, while trimodular RCCX haplotypes have a frequency of about 14% [7]. The *CYP21A2 *gene and *CYP21A1P *pseudogene each contain 10 exons spaced over 3.1 Kb, their nucleotide sequences are 98% identical in exons and approximately 96% identical in introns [8]. Intergenic recombinations are responsible for 95% of the mutations associated with 21-hydroxylase deficiency; the remaining 5% of mutations do not appear to be the result of gene conversion events [9,10]. Among the intergenic recombinations, approximately 75% is represented by mutations normally present in the pseudogene and possibly transferred to the functional gene by microconversion events [11]. The remaining 20%–25% of mutations are *CYP21A2 *gene deletions or *CYP21A1P/CYP21A2 *chimeric genes. In fact, the 26 or 32 Kb deletion (depending on whether C4B is the long or short gene), involving the 3' end of *CYP21A1P*, all of the *C4B *gene, and the 5' end of *CYP21A2*, produces a single non functional chimeric gene with its 5' and 3' ends corresponding to *CYP21A1P *and *CYP21A2*, respectively [1,11]. To date, five different chimeric *CYP21A1P/CYP21A2 *genes have been found and characterized in recent studies [12-16]. In this paper, we describe a new *CYP21A1P/CYP21A2 *chimeric gene (*CH-6*) found in an Italian patient suffering from a severe form of CAH.

## Methods

### Patients

The patient is the first daughter of non consanguineous parents of Italian origin. No family history of CAH, of virilisation in female family members or of impaired fertility was reported. Pregnancy and delivery were uneventful and birth weight and length were normal (3400 g, 51 cm). Diagnosis of CAH was suspected due to genital virilisation (Prader IV). Thus, hormonal evaluation and kariotype were requested. Plasma levels of Na and K were 124 mEq/L and 6.2 mEq/L, respectively, while 17-OHP plasma value, on fourth day of life, was 223 nmol/L. Kariotype showed a normal female pattern (46, XX). Therapy with betamethasone and fluorohydrocortisone was started on the 11^th ^day. No vomiting or other clinical signs of salt wasting were shown before onset of substitutive therapy.

Clitoromegaly was corrected at 4 yr of age and hydrocortisone therapy was started at 6 yr. The patient showed a normal pubertal development with menarche at 14 yr and she underwent vaginoplasty at 17 yr. She married at 26 yr with a healthy Italian male and she was pregnant at 29 yr.

### Molecular analysis of CYP21A2 gene

Informed consent for genetic study was obtained, it was in compliance with the Helsinki Declaration and was approved by Catholic University Ethics Committee (Reference Number: P6242008).

Genomic DNA was isolated from peripheral blood samples using High Pure PCR Template Preparation Kits (Roche Diagnostic, USA), quantified by spectrophotometer at 260 nm and stored at -20°C until use. In addition, since the patient decided to undergo an amniocentesis at 18^th ^week of gestation, a DNA sample was extracted from amniotic fluid cells.

Two genomic DNA fragments, spanning from the 5' region to exon 6 (FRAG 1) and from exon 6 to 3'region (FRAG 2) of the active *CYP21A2 *gene, were amplified using two sets of specific primers, as previously described [17]. In addition, the *CYP21A2 *promoter region was amplified using the primers: PROM-F 5'-gca ggg act gcc att ttc tc-3' (nucleotides 87034–87053; GeneBank accession AL049547) and PROM-R 5'-agc agg gag tag tct ccc aag-3'(nucleotides 85935–85955; GeneBank accession AL049547).

PCR-amplified fragments were directly sequenced using BigDye Terminator Cycle Sequencing kit v3.1(Applied Biosystems, USA) and ABI 3100 Avant Genetic Analyser (Applied Biosystems, USA) according to manufacturer's instructions.

The results were analysed using the SeqScape v2.5 software package (Applied Biosystems, USA). The *CYP21A2 *sequence references were: NCBI-AL049547 and NC-000006.

### HLA typing

HLA-B and HLA-DRB1 genotypes were determined using a semi-automated, commercially available reverse dot-blot method (Inno-lipa, Innogenetics, Gent, Belgium), according to the manufacturer's instructions. Reaction patterns were interpreted using INNO-LiPA software.

### CYP21/C4 haplotyping

Leukocyte DNA was digested with TaqI restriction enzyme and Southern blotting studies were conducted as previously described [18]. Blots were probed with a mixture of two fragments: a 500-bp BamHI-KpnI 5' fragment of C4 cDNA (pAT-A clone) and a 3.1-Kb genomic EcoRI-BamHI fragment of the 5.5-Kb BglII-BamHI fragment encompassing the entire *CYP21A2 *gene cloned in the BamHI site of bluscript SK+ plasmid [18,19].

Band ratios were measured by laser densitometry (Ultroscan XL laser densitometer; Pharmacia LKB).

### Isolation of CYP21A1P/CYP21A2 chimeric gene

In order to isolate the chimeric *CYP21A1P/CYP21A2 *allele, two different strategies were used. The first was an allele specific PCR performed using the primers: PROMPS 5'-cca ggt cgg ggc gga cac cc-3' (nucleotides 433–452; GeneBank accession NC_000006.10 [region: 32.080.697...32.084.739]) and PROM-R (nucleotides 85935–85955; GeneBank accession AL049547). The PROMPS forward primer was specific for a *CYP21A1P *pseudogene 5' sequence while the reverse PROM-R primer was specific for exon 3 of the *CYP21A2 *active gene. The PCR fragment, spanning from the 5' region to exon 3 of the chimeric gene, was directly sequenced.

The second strategy was the amplification (Expand Long Template PCR System, Roche Diagnostic, USA), using CYP779f and Tena36F2 primers, of a 6.2Kb fragment encompassing the 5'-end of the *CYP21A2 *gene and exon 36 of the *TNXB *gene [20]. After amplification, 700 ng of the PCR product was incubated at 65°C for 2 h with 10 U of Taq I restriction enzyme. The completely digested PCR product was analyzed by electrophoresis on a 1.2% agarose gel to evaluate the presence of the 3.7- and 3.2-Kb fragments produced by the active *CYP21A2 *gene and the chimeric *CYP21A1P/CYP21A2 *gene, respectively [20]. The 3.2 Kb fragment including the whole *CYP21A1P/CYP21A2 *chimera [being generated by the Taq I cutting at 86870 position (GeneBank accession AL049547), corresponding to -209 C nucleotide in the *CYP21A1P *promoter region, and at 83662 position (GeneBank accession AL049547) within 5' region of *TNXB *gene] was isolated from agarose gel (QIAEX II Gel Extraction Kit, Qiagen, Hilden, Germany) and sequenced using internal primers (available on request). In order to confirm the obtained results the 6.2 kb fragment was cloned into the pGEM-T vector system (Promega, Madison, WI, USA) and the identified clone was directly used as template for DNA sequencing.

## Results

### Molecular analysis of CYP21A2 gene

The *CYP21A2 *sequencing analysis showed that the patient was homozygote for the g.655C/A>G mutation in intron 2 and heterozygote for the p.P30L missense mutation in exon 1. In addition, the promoter sequence revealed the presence, in heterozygosis, of 13 variants (g.-306G>C, g.-295T>C, g.-294A>C, g.-283A>G, g.-281T>G, g.-210T>C, g.-199C>T, g.-190insT, g.-126C>T, g.-113G>A, g.-110T>C, g.-103A>G and g.-4C>T) which are generally the result of microconversion events between gene and pseudogene. In this first sequencing analysis step, we were unable to perform the complete nucleotide analysis of intron 2 since the electropherogram showed various frameshift mutations resulting in two overlapping sequences.

The same problem was present when the fetus'*CYP21A2 *sequence was analyzed. However, the results showed that the fetus' DNA carried, in heterozigiosis, both the g.656C/A>G and p.P30L mutations in addition to the 13 variants in the promoter region.

Finally, the patient's husband resulted wild type for the *CYP21A2 *sequencing analysis.

### HLA typing

The results of HLA-DRB and HLA-B genotypes were as follows: patient: HLA-B*35/44 HLA-DRB1*01/13; husband: HLA-B*15/51 HLA-DRB1*11/13; male fetus: HLA-B*15/44 HLA-DRB1*01/13. These results confirmed the correct segregation of HLA loci.

### CYP21/C4 haplotyping

**Figure 1:**
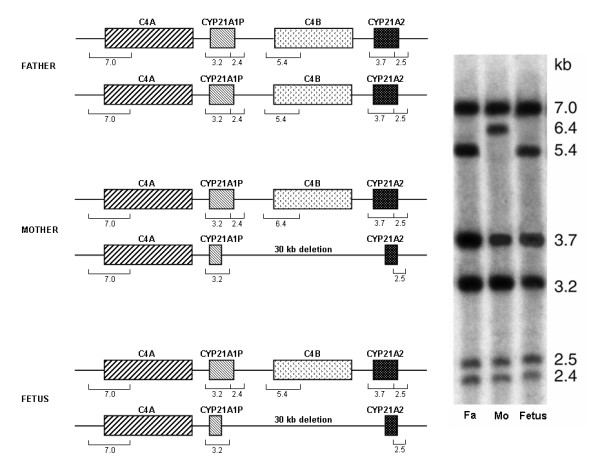
**Southern blot analysis**. Taq I restriction patterns of genomic DNA hybridized to a mixture of the *CYP21A2 *and *C4 *cDNA probes. Fa: father, Mo: mother. For pattern analysis see the text in results section.

The Southern blot results are shown in Fig. 1 and were interpreted as follows: 1) the fetus' father (lane 1) carried two bimodular chromosomes with a total of two *CYP21A2 *genes (3.7 kb and 2.5 kb bands), two *CYP21A1P *pseudogenes (3.2 kb and 2.4 kb bands), two long telomeric C4 genes(7.0 kb band) and two short non-telomeric C4 genes (5.4 kb band); the patient (lane 2) carried a chromosome with a normal bimodular structure (one *CYP21A2 *gene, one *CYP21A1P *pseudogene, one long telomeric C4 gene and one long non-telomeric C4 gene corresponding to 6.4 kb band) and a chromosome with the classic 30 Kb deletion (as shown by decreased intensities of 6.4 kb band, corresponding to the C4B gene, and of 3.7 kb and 2.4 kb bands corresponding to the 5' section of the *CYP21A2 *active gene and to the 3' fragment of the *CYP21A1P *pseudogene, respectively); finally, the fetus (lane 3) inherited from the father a wild type bimodular chromosome with a short non-telomeric *C4 *gene and from the mother the chromosome with the 30 kb deletion (as shown by the absence of the 6.4 kb band and by the decreased intensities of 3.7 kb and 2.4 kb bands).

### Sequence of CYP21A1P/CYP21A2 chimeric gene

**Figure 2:**
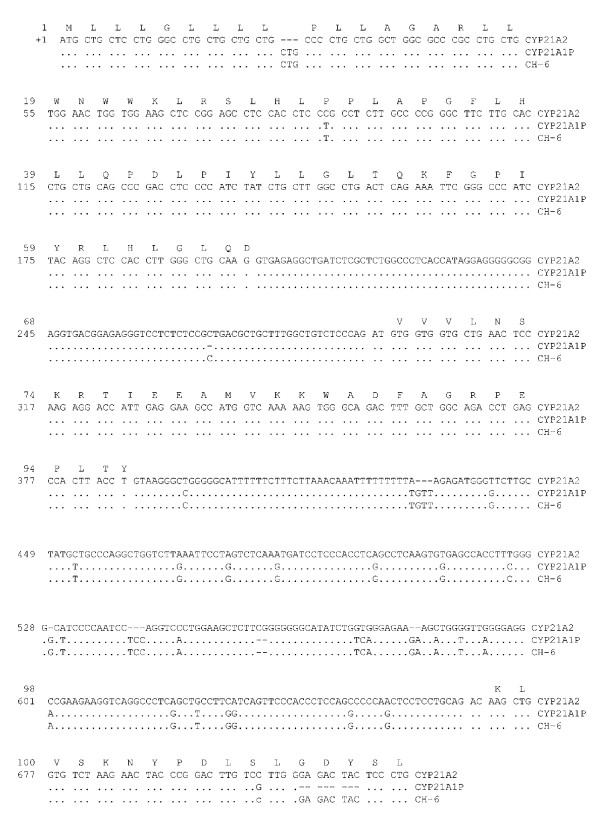
**Sequence of the new chimeric gene**. Sequence of *CH-6 *chimera from +1 nucleotide (exon 1) to +721 nucleotide (exon 3). Nucleotides were numbered starting from ATG. The lines showed: the amino acid sequence of *CYP21A2 *gene (M13936), the *CYP21A2 *nucleotide sequence (M13936), the *CYP21A1P *nucleotide sequence (M13935) and the *CH-6 *nucleotide sequence. Only nucleotide differences between *CYP21A1P *or *CH-6 *and *CYP21A2 *active gene are shown under corresponding nucleotides in *CYP21A2 *gene.

The 6.2 Kb long PCR digested with TaqI enzyme[20] combined with cloning and sequencing of 3.2 Kb fragment allowed to prove the existence of the new chimeric gene. In fact, the sequencing analysis showed that *CH-6 *gene have a *CYP21A1P-like *sequence up to 656 nucleotide of intron 2 while the remaining exons/intons have a wild type *CYP21A2 *sequence (Fig. 2).

## Discussion

The chimeric *CYP21A1P/CYP21A2 *gene is the consequence of the 26 or 32 Kb deletion including the complete *XA*, *RP2*, and *C4B *genes and the partial sequences of *CYP21A1P *and *CYP21A2 *genes [20].

The possible cause of the chimera formation is the presence of specific sequences, such as *Chi*-like and tandem-repetitive minisatellite consensus, which play a role in promoting genetic recombination [21]. In particular, it has been pointed out that the *chi*-like sequence GCTGGGG is present several times in the intronic regions of *CYP21A2 *and *CYP21A1P *genes [22]. However, the high degree of sequence homology of *RP1-C4A-CYP21A1P-XA-RP2-C4B-CYP21A2-TNXB *genes arranged in tandem, seems to be the most probable way to increase the chance of misalignment at meiosis to generate genetic recombination in the 6p21.3 chromosome area [23].

**Figure 3:**
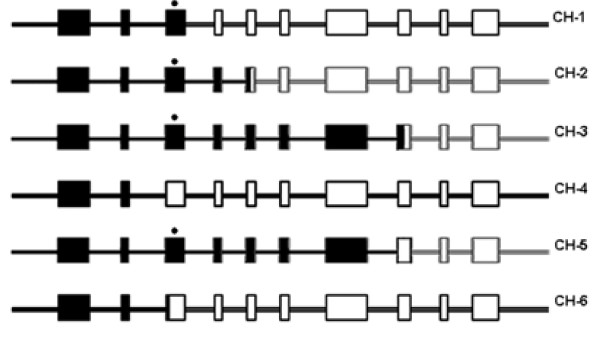
***CYP21A1P/CYP21A2 *chimeric genes**. *CH-1, CH-2, CH-3, CH-4, CH-5 *and *CH-6 *represent six distinct chimeric *CYP21A1P/CYP21A2 *genes as described in the text. The black and white regions represent the non-functional *CYP21A1P *and the functional *CYP21A2 *sequences, respectively. The eight nucleotide deletion in exon 3 is marked by an asterisk.

To date, five different chimeric *CYP21A1P/CYP21A2 *genes have been found and characterized [12-16] (Fig. 3). Three of them, found in ethnic Chinese (Taiwanese), are named *CH-1*, *CH-2*, and *CH-3 *[12-14]. In these molecules, the deletion g.707–724delGAGACTAC in exon 3 leads to a frameshift mutation which forms a TGA stop codon downstream 830 nucleotide producing a truncated protein.

*CH-4 *chimera was found in a Caucasian NC-CAH patient [15] and it is probably the same one earlier reported by Killeen et al. [24]. *CH-4 *junction site was located between the end of exon 1 and the beginning of intron 2, consequently, the resulting hybrid gene differs from the functional gene only for the presence of two deleterious mutations: the weak promoter region of the pseudogene and the p.P30L missense mutation in exon 1. Finally, *CH-5 *chimera, associated with the HLA-B47, DR7 haplotype, was described by White et al. [16] and results very common among CAH patients of Caucasian origin.

In this paper, we report a new *CYP21A1P/CYP21A2 *chimera (*CH-6*) found in an Italian woman suffering from salt-wasting form of CAH. The hybrid junction site was located between the end of intron 2 pseudogene, after the g.656C/A>G mutation, and the beginning of exon 3, before the 8 bp deletion. Consequently, *CH-6 *carries three mutations: the weak pseudogene promoter region, the p.P30L in exon 1 and the g.655C/A>G splice mutation in intron 2 (Fig. 3). Since the patient carried a second *CYP21A2 *allele with the g.656C/A>G mutation, her clinical severe phenotype was determined by the homozygote status for this mutation. Unfortunately, it was not possible to perform a more extensive familiar genetic study and therefore we can not determine if the patient inherited the new *CH-6 *chimeric gene from one of her parents.

However, our molecular CAH prenatal diagnosis showed that the fetus inherited the new chimeric gene from his mother and a wild type *CYP21A2 *allele from his father, as also confirmed by HLA typing.

## Conclusion

In conclusion, we describe a new *CYP21A1P/CYP21A2 *chimera (*CH-6*), associated with the HLA-B15, DR13 haplotype, in a young Italian CAH patient.

## Competing interests

The authors declare that they have no competing interests.

The study was carried out by means of funding of Institute of Biochemistry and Clinical Biochemistry, Catholic University of Rome.

## Authors' contributions

PC carried out the molecular genetics studies, participated in the sequence alignment and drafted the manuscript.

EM participated in the molecular genetics study and in the sequence alignment.

AM participated in the molecular genetics studies and in the sequence alignment.

EG participated in the molecular genetic studies.

CZ participated in the design of the study and in its coordination.

VT performed clinical study and participated in the design of the study and in its coordination.

EC participated in the design of the study and drafted the manuscript.

All authors read and approved the final manuscript.

## Pre-publication history

The pre-publication history for this paper can be accessed here:


